# “Click for Closer Care”: A Content Analysis of Community Pharmacy Websites in Four Countries

**DOI:** 10.2196/jmir.6899

**Published:** 2017-06-14

**Authors:** Sandra Zwier

**Affiliations:** ^1^ Amsterdam School of Communication Research ASCoR Department of Communication Science University of Amsterdam Amsterdam Netherlands

**Keywords:** community pharmacy services, pharmaceutical services, online pharmacies, marketing of health services, commerce, pharmacy ethics

## Abstract

**Background:**

Combinations of professional and commercial communication are typically very controversial, particularly in health care communication on the Internet. Websites of licensed community pharmacies on the other hand tend to raise remarkably little controversy, although they typically contain controversial combinations of clinical and commercial services previously unprecedented in professional health care communication.

**Objective:**

The aim of this study was to fill the void of knowledge about the combination of clinical and commercial services presented on the websites of licensed community pharmacies.

**Methods:**

A content analysis of clinical and commercial services presented in a random sample of 200 licensed community pharmacy websites from Great Britain, the Netherlands, the Canadian provinces British Columbia and Manitoba, and the Australian states New South Wales and Western Australia was conducted.

**Results:**

The top five specific services mentioned on the community pharmacy websites were cosmetic products (126/200, 63.0%), medication refill request options (124/200, 62.0%), over-the-counter medicine (115/200, 57.5%), complementary and alternative medicine (107/200, 53.5%), and home medical aids (98/200, 49.0%). On average, 72.5% (145/200) of the community pharmacy websites across the 4 countries included a combination of clinical and commercial services. A combination of clinical and commercial services was more often present on chain pharmacy websites (120/147, 82.8%) than single pharmacy websites (25/53, 47%; *P*<.001), and most often on the Canadian community pharmacy websites, followed by the Australian, British, and Dutch pharmacy websites, respectively (*P*<.02). Furthermore, more than half of the pharmacies’ homepages contained a combination of clinical and commercial images (107/200, 53.5%), and almost half of the homepage menus contained a combination of clinical and commercial items (99/200, 49.5%). The latter were, again, more common on chain pharmacy than single pharmacy websites (*P*<.001), with significant differences between countries (*P*<.001).

**Conclusions:**

A considerable share of websites of licensed community pharmacies in Great Britain, the Netherlands, Canada, and Australia combine clinical services with commercial services. Previous research into the presence of a combination of commercial and professional services suggests that such a combination may lead to increased interest in commercial services that may be unnecessary or inappropriate to patients’ health.

## Introduction

### Combinations of Professional and Commercial Health Communication

Combinations of professional and commercial communication are typically very controversial, particularly in health care, where vulnerable patient groups may become interested in goods or services that may be unnecessary, inappropriate, or even dangerous to their health [1-3]. That is why such a combination often gives rise to calls for stringent regulatory action, meanwhile generating significant volumes of research. Examples include a combination of clinical and commercial communication in cosmetic surgery [4,5], medical tourism [6,7], and robotic surgery [8,9]. Recent years have also witnessed a lot of alarm surrounding the surge of illicit online pharmacies that sell prescription drugs without a physician’s prescription, and are notorious for the use of hard-line marketing techniques such as mail spamming and search engine contamination [10-12]. On the other hand, remarkably little controversy or research surrounds the websites of regular, licensed community pharmacies. However, as we will argue, community pharmacy websites are gaining increasing importance as online “health care hubs” where patient consumers may submit electronic prescriptions, order prescription and over-the-counter medication, consult pharmacists about a chronic condition, obtain a prescription for a minor ailment, download software to monitor their health, and order personal care and household products, among others. They are also one of the major mainstream venues where professional clinical and commercial communication come together on the Internet [13-17], and this could be a potential minefield where many patients can easily confuse commercial communication for professional advice.

This study aimed to fill the currently large void in knowledge about the contents of licensed community pharmacy websites, guided by the following research question: *To what extent do present-day licensed community pharmacy websites combine clinical and commercial communication*? This question was investigated through a systematic content analysis of licensed community pharmacy websites from Australia, Canada, Great Britain, and the Netherlands. Besides gauging a picture of the combination of clinical and commercial content on officially licensed community pharmacies’ websites in a number of Western countries nowadays, this study also provided an overview of the range of clinical and commercial services put forward on present-day community pharmacy websites, guided by the subquestions: *What types of clinical services are mentioned on present-day licensed community pharmacy websites*? and *What types of commercial services are mentioned on present-day licensed community pharmacy websites*? Finally, although cross-national comparisons were not the study’s aim, the study could also answer the subquestion: *What cross-national differences exist in the types of clinical and commercial services mentioned on community pharmacy websites*? Implications for public health education are also discussed.

### Background

The ideal clinical professional stays away from involvement in commercial communication, or so it seems. Professional clinical associations often promote straight bans or use highly restrictive codes of conduct for members’ commercial communication, sometimes against pressure from government administrations to loosen their regulations. The American Federal Trade Commission (FTC), for instance, has fought a lengthy statutory battle against the American Medical Association for restricting free competition through its persistent ban on physician advertising. This was later followed up by comparable cases of the FTC against other professional associations for restricting advertising, such as dentists, optometrists, accountants, and lawyers [18]. Also for instance the Israel Medical Association upheld a long-lasting public doctors’ strike after the Israeli government proposed a loosening of the absolute ban on physician advertising in the country [19]. Professional clinical providers also typically have strong adverse attitudes toward marketing and advertising their services [20-22]. On the Internet, this is mirrored in the continuous effort by diverse medical and health associations to establish quality standards for professional health information. This can be seen with examples such as the “Health on the Net Foundation Code of Conduct” (HONcode) which states that “advertising should be clearly distinguished from editorial content.” Similarly, the guidelines of the American Medical Association state that on the Internet “advertising and commercial sponsorship must not influence any editorial content and advertising must be easily discernible from editorial content” [23-25].

The principal argument against clinical professionals’ involvement with commercial communication revolves around a perceived conflict between commercial communication on the one hand and the clinical profession’s “fiduciary responsibility” on the other, that is, the duty to prioritize and protect patients’ interests at all times [26,27]. The premise is that trust is a central feature of clinical professionals’ relationship with their patients, and commercial communication could compromise this trust [3,20]. In persuasive communication literature, the trust that people have in the accuracy and reliability of an information source is referred to as “source credibility.” It is one of the oldest [28] and most frequently studied factors in persuasive communication research [29]. Research exploring the role of source credibility in the acceptance of online health information often shows that online health information is seen as more credible and convincing when the source is a perceived expert, such as a physician or professional health organization, than a lay person [30-34]. A commercial service mentioned on a physician or hospital’s website will thus be likely to be seen as a higher-quality service than the same service featured on, for instance, the website of a brand or an online retailer, such as Amazon.

### Clinical and Commercial Services in Community Pharmacy

This study focused on the combination of clinical and commercial communication on community pharmacy websites. On the basis of the existing strong norms against combining clinical and commercial communication in the clinical professions, most people would be at least surprised, and possibly dismayed, if their family physician’s website would promote a branded pain reliever or line of mascaras. However, it is not uncommon to see this on a community pharmacy website. For example, the website of Service Apotheek, one of the largest community pharmacy chains in the Netherlands, mentions clinical consultation services for patients with chronic conditions such as diabetes and chronic obstructive pulmonary disease (COPD). At the same time, the website presents promotion for a branded facial cream that is claimed to “fill the skin with youth” and a slimming belt that will allegedly “redefine the body contours instantly.” Such a combination of clinical and commercial communication can be found in community pharmacies across the world today, from Australia [35], to Kenya [36], Peru [37], and the Vatican [38].

Given today’s proliferation of commercial communication in community pharmacies, it may be assumed that a community pharmacy is largely evolving into a commercial retail business, with little in the way of an enduring role in the provision of clinical health care. Nonetheless, this is only one side of the coin. Community pharmacies’ clinical care activities largely waned during the course of the 20^th^century, when the “golden age of doctoring” was in its heydays and pharmacists’ role largely hinged on those of physicians [15,39,40]. As one pharmacist said about his time in a community pharmacy in the 1970s, “We were discouraged from talking to patients for fear we would disrupt the doctor-patient relationship” [41]. Pharmacies since, however, have taken on a more patient-centered role and are becoming increasingly more involved in patient therapy [42,43]. Moreover, in recent years many countries such as China, Great Britain, New Zealand, and Switzerland have installed programs for clinical services to be delivered by community pharmacies, in an effort to control the steep rise in costs of clinical care delivered by family physicians and hospitals [44-46]. Community pharmacies’ clinical role thus has risen “like a phoenix from the ashes” [39]. Present-day clinical services offered through community pharmacies may range from patient education, to consultations for patients with chronic conditions, to primary care provision, to actual prescribing [39,47-50]. For instance, Walgreens pharmacy in the United States currently has 400 so-called “health care clinics” across the country where the public may obtain treatment for minor illnesses. Similarly, Boots pharmacy in the United Kingdom features a number of “online clinics” where people can obtain prescription-only medication without going to the doctor for conditions such as erectile dysfunction, period delay, and malaria prevention.

### Study Aims

All in all, developments in community pharmacies over the last few years have led to the presence of a combination of clinical and commercial services that were previously unprecedented in the domain of professional clinical care. To what extent this is mirrored on community pharmacy websites however is unknown. Extant research on community pharmacy websites is scant, with very few exceptions [51,52]. Thus, this study aimed to fill the void in knowledge by documenting the types of clinical and commercial services present on licensed community pharmacies’ websites in 4 Western countries and the extent to which the websites contain a combination of clinical and commercial services.

## Methods

### Content Analysis

A systematic content analysis was conducted to allow for an unobtrusive observation of representative samples of licensed community pharmacies’ websites and their contents. To strengthen the generalizability of the findings beyond the specific context of one particular country, community pharmacy websites from 4 different countries and different continents were included in the study, namely from Great Britain, the Netherlands, Canada, and Australia. The choice for these 4 countries was in part based on practical reasons of the researchers’ command of the languages. Furthermore, low and middle-income countries were not included because pharmacy legislation may often be fragmented with limited enforcement of regulations in these countries [53], and this would significantly hinder identification of the population of licensed pharmacies in these countries. Finally, the United States and New Zealand were excluded because they currently are the only two countries in the world where direct-to-consumer advertising (DTCA) of prescription medication is permitted [3]. This exceptional regulatory status may restrict the generalizability of the findings regarding commercial content on community pharmacy websites, to most other countries in the world where DTCA of pharmaceuticals is prohibited. All in all, this study involved a content analysis of community pharmacy websites from Great Britain, the Netherlands, Canada, and Australia as countries with highly institutionalized and enforced pharmacy legislation and a ban on DTCA for medical drugs. Without making unsubstantiated claims regarding the representativeness of these 4 countries for community pharmacy websites worldwide, this likely renders a conservative rather than exaggerated picture of combinations of clinical and commercial services on community pharmacy websites worldwide nowadays.

### Sample

#### Sampling Method

The sample consisted of 200 licensed community pharmacy websites: 50 from Great Britain, 50 from The Netherlands, 50 from the Canadian provinces British Columbia and Manitoba, and 50 from the Australian states New South Wales and Western Australia.

The sample of British community pharmacies was randomly drawn from a directory of all 14,437 licensed community pharmacies from the British General Pharmaceutical Council, and the sample of Dutch pharmacies from a directory of all 1981 licensed pharmacies in the Netherlands from the Dutch Healthcare Inspectorate. Community pharmacy licensing and directories in Australia and Canada are organized by the given state or province, which is why the sampling of community pharmacies proceeded with the use of the directories of two states or provinces per country. For Canada, 25 community pharmacy websites were drawn from the directory of the 1256 licensed community pharmacies of the College of Pharmacists of British Columbia and 25 from the 415 registered pharmacies by the College of Pharmacists of Manitoba. For Australia, 25 community pharmacy websites were drawn from the directory of the 2514 licensed community pharmacies by the Pharmacy Council of New South Wales and 25 from the 637 licensed pharmacies by the Pharmacy Registration Board of Western Australia.

Since this study endeavored to study community pharmacy websites, community pharmacies in the directories without a (working) website were excluded from the sample; this concerned 33.3% (67/200) community pharmacies of the original selection (26 Australian, 13 British, 28 Canadian, and 0 Dutch original selections). Also, the pharmacy departments of mass merchants and health centers had to be excluded. This is because the websites of these merchants and centers do not allow a valid separation between services offered through the pharmacy department and services offered through other departments, such as those of family physicians or a nurse on a health care center’s website or banking or petrol services on a retail centers’ website. Inclusion of these websites thus would have led to a significant overestimation of combinations of clinical and commercial services on community pharmacy websites. This concerned 8.5% (17/200 community pharmacies of the original selection (4 Australian, 4 British, 7 Canadian, and 2 Dutch original selections). When a pharmacy in the random selection had to be excluded from the sample, the next one in the directory was always chosen.

#### Sample Characteristics

Table 1 provides an overview of the share of chain pharmacies versus single pharmacies per country in the sample, with the former defined as community pharmacies belonging to a group of four or more community pharmacies that operate under a joint brand name [54]. It can be seen that almost three quarters (73.0%; 147/200) of the community pharmacies in the sample operated under a chain pharmacy retail brand name, with little variation across countries.

**Table 1:** Share of single and chain community pharmacies in the sample.

Pharmacy business type	Community pharmacies				
	Australia, N=50	Great Britain, N=50	Canada, N=50	Netherlands, N=50	All, N=200
Single pharmacy, n (%)	15 (30)	10 (20)	12 (24)	16 (32)	53 (27.0)
Chain pharmacy^a^, n (%)	35 (70)	40 (80)	38 (76)	34 (68)	147 (73.0)

^a^Chain pharmacies are community pharmacies belonging to a group of four or more community pharmacies that operate under a joint brand name and often participate in one or more centralized programs [55].

Considering the present study’s aims, it is of relevance to note that community pharmacies operating under a chain pharmacy retailer name often participate in one or more centralized programs, such as supply chains, pharmacist training schemes, or store brands. This could impact the variation in the services mentioned on the websites. However, the extent to which individual pharmacies partake in the pharmacy chain’s centralized programs varies between, as well as within, pharmacy chains, while individual pharmacies may also include their own local services on the website. For instance, the Boots community pharmacy from the town of St Austell in the United Kingdom in our sample listed 10 services on its website, whereas the Boots pharmacy from the British town of Peebles in our sample listed only 2 services. That is why each community pharmacy website in the sample was coded separately and often contributed to variation in the data.

### Coding Instrument and Procedure

**Figure 1:**
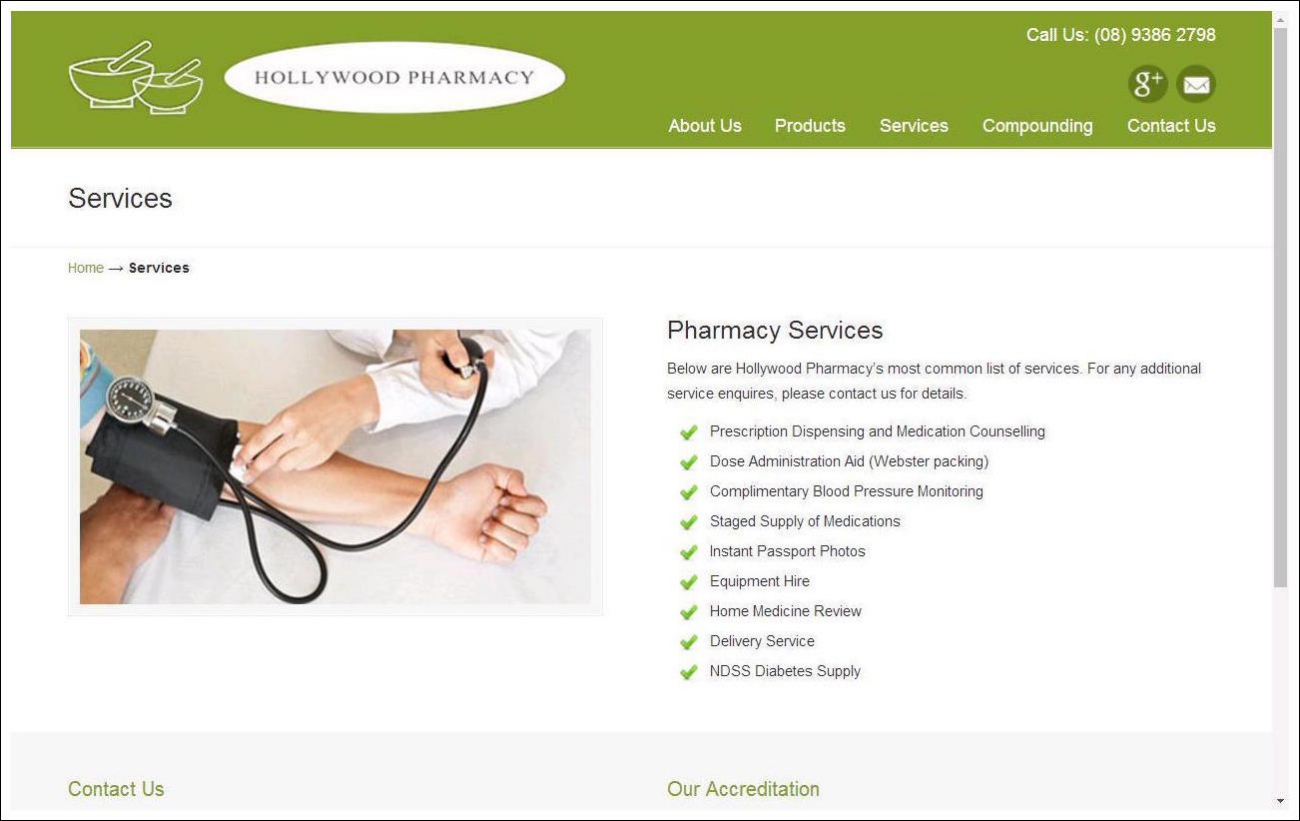
Screenshot of website Hollywood Pharmacy, Nedlands (Australia), taken on March 29, 2017.

**Figure 2:**
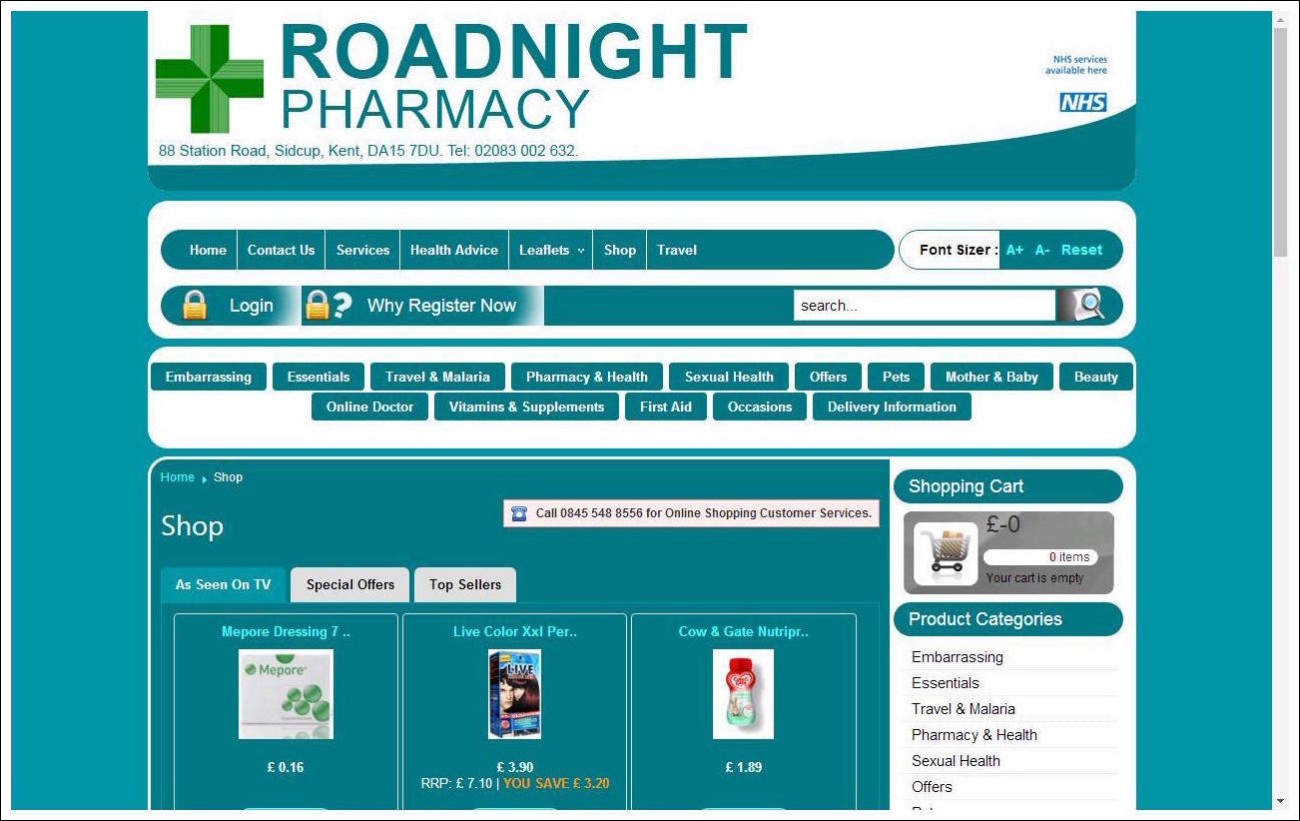
Screenshot of website Roadnight Pharmacy, Sidcup (Great Britain), taken on March 29, 2017.

**Figure 3:**
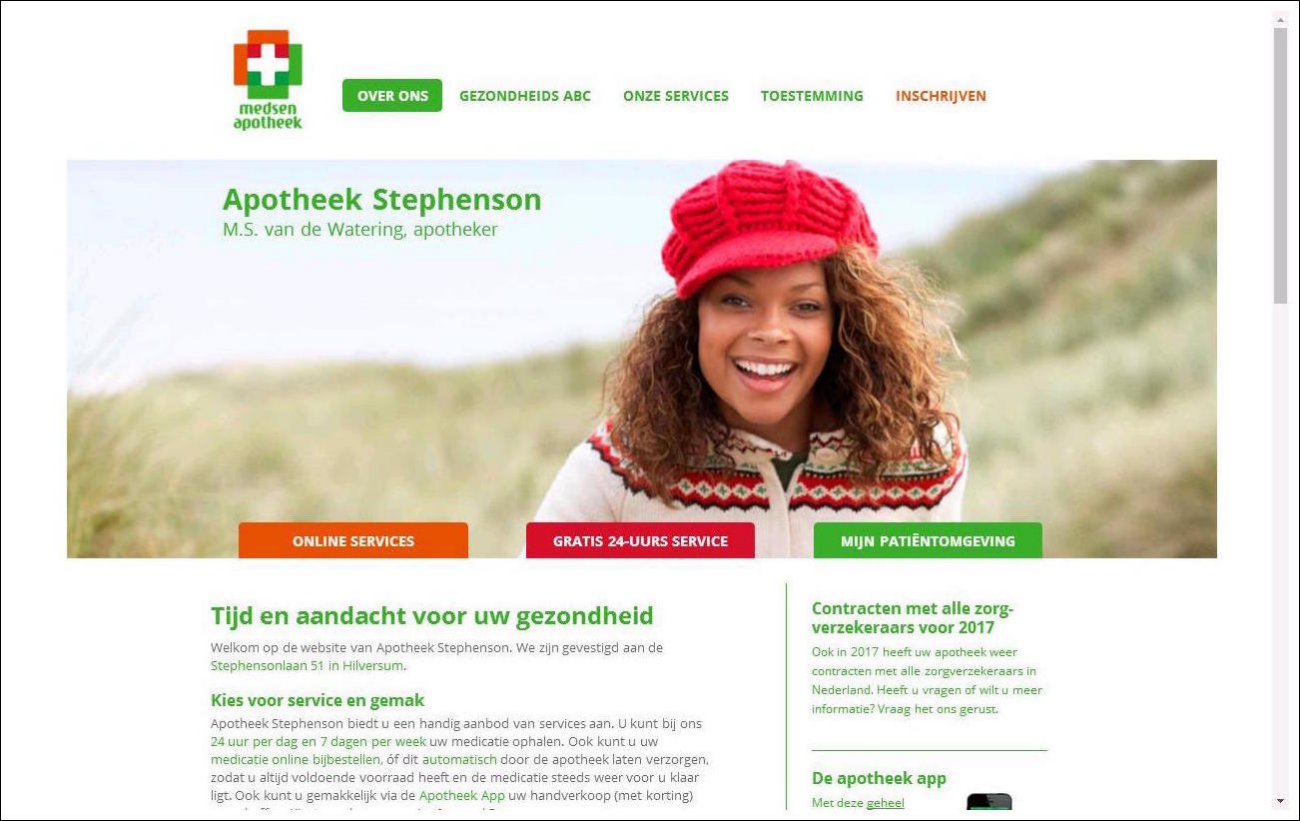
Screenshot of website Medsen pharmacy, Hilversum (Netherlands), taken on March 29, 2017.

**Figure 4:**
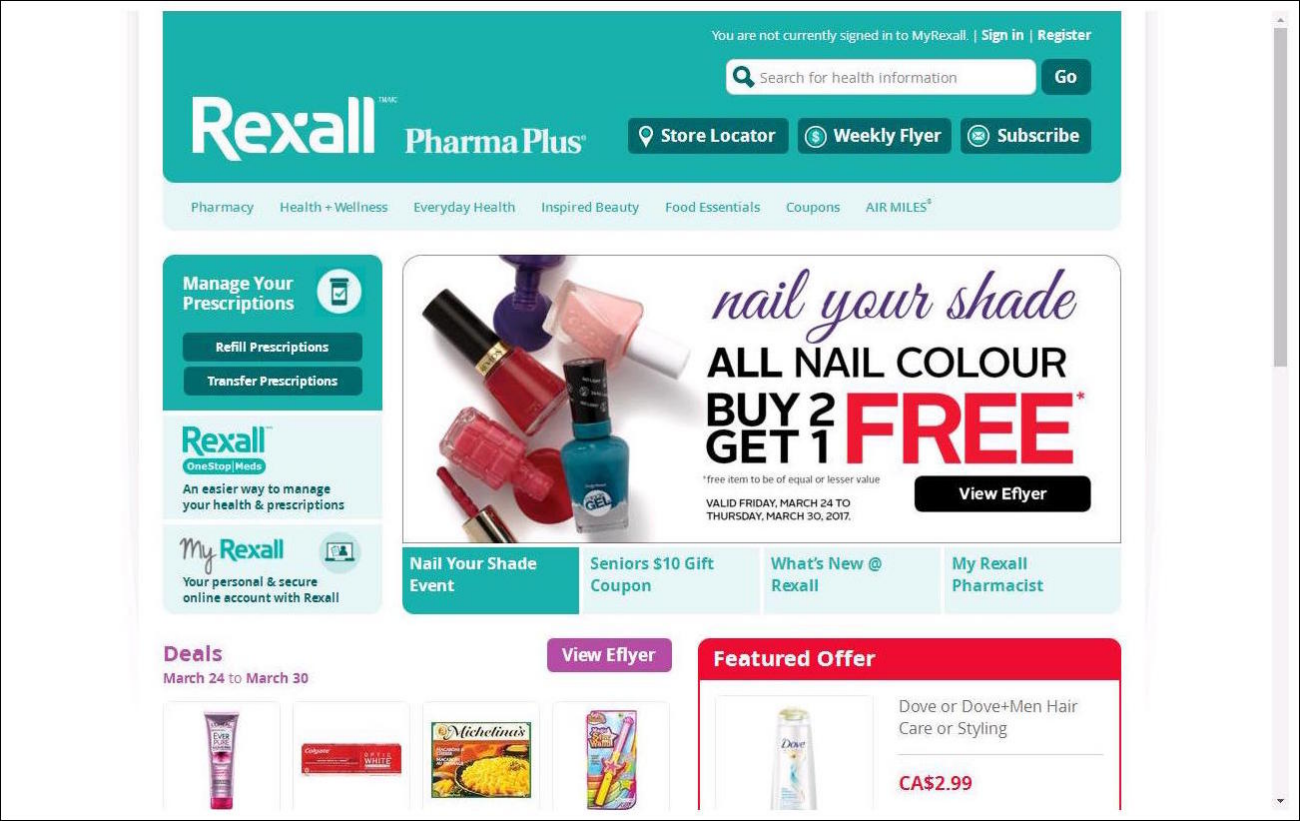
Screenshot of website Rexall pharmacy (Canada), taken on March 29, 2017.

Figures 1-4 show screenshots of community pharmacy websites from the sample. Each community pharmacy website in the sample obtained a binary code for the presence or absence of specific categories of clinical and commercial services on the website. Since a number of discrete products and services are concerned that obtain their meaning from the trade’s definition of these products/services, measures of intersubjective agreement were not deemed relevant here and the coding was done by a single author. Below is a rationale for and overview of the coding categories employed.

#### Clinical and Commercial Services

##### Clinical Services

Clinical services were defined as clinical and pharmaceutical goods and services that are typically restricted to qualified pharmacists and other health care professionals. Three categories of clinical goods and services were distinguished in the coding, based on how current community pharmacy commonly defines its own roles [39,41-45]. The first of these is community pharmacy’s traditional role of supervising the dispensing of medication. As community pharmacy in more recent years has expanded beyond the traditional function of dispensing and distributing medicine to more patient-based care, a second commonly recognized role of community pharmacy is disease detection and prevention. This includes services to maintain health such as vaccinations and stop smoking counseling, but also the timely detection of conditions such as hypertension or diabetes to combat the adverse consequences for patients more effectively. Third and finally, community pharmacy assumes a role in consulting patients about the effective and safe use of medication to achieve optimal patient outcomes, particularly for patients with chronic conditions such as hypertension, asthma, or diabetes [39,41-45].

In the coding procedure, each community pharmacy website was coded for the presence of the 3 above-mentioned roles of community pharmacy, specifically: *Dispensing services*, that is, services surrounding the dispensing of prescription medication (eg, dispensing of prescription medication, dispensing hormone replacement therapy), *Disease prevention and detection services*, that is, methods for diagnosing conditions and avoiding illness (eg, diabetes test, flu vaccinations), and *Clinical consultations*, that is, consultations for patients with specific conditions (eg, consultations for asthma patients, diabetes patients). Specific subcategories within these 3 coding categories, such as the testing of blood pressure, cholesterol, diabetes, and so on, were derived from the categorizations on the pharmacy websites themselves. Clinical services that typically belong to other professional health care domains such as veterinarian, optometric, or dental care services were excluded from coding. The screenshot of the website of Hollywood pharmacy from the town of Nedlands in Australia in Figure 1 illustrates the 3 categories of clinical services. Specifically, we see that the list of pharmacy services on this website includes dispensing services (“prescription dispensing,” “dose administration aid”), disease detection and prevention services (“complementary blood pressure monitoring”), and clinical consultation services (“home medicine review,” a consultation to review a patient’s medication use to check for possible overlaps or interactions).

##### Commercial Services

Commercial services were defined as all services mentioned on the community pharmacy websites that are not typically restricted to licensed health care professionals. Again, 3 categories of commercial goods and services were distinguished. The first category reflects pharmacies’ traditional role of the sale of health-related goods and services such as cold and cough medicine or pain relievers to ease and combat ills. The second category, the sale of nonhealth services, reflects the rise of the “drugstore” and “convenience store” concept in community pharmacy with an expanded focus on the sale of a range of everyday products such as cosmetics, snacks, and cleaning supplies. Finally, the third category reflects the move away from the brick-and-mortar commercial sale only, to an e-commerce model with the integration of a Web shop in community pharmacy websites.

In the coding procedure, each community pharmacy website was coded for the presence of the above-mentioned commercial roles of community pharmacy, specifically: *Health services*, specifically the sale of over-the-counter medicine (eg, pain relievers, first aid kits), complementary and alternative medicine (eg, homeopathic products, aromatherapy), diet products (eg, slimming shakes, gluten-free products), and home medical aids (eg, mattress covers, shower stools). *Non-health services*, specifically cosmetic products (eg, soaps, make-up products), food and drinks (eg, candy, snacks), and other goods or services (eg, greeting cards, pet products, kitchen appliances, photo services). *E-commerce*, that is, options to purchase services online through the pharmacy website, often through a Web shop. Again, the specific subcategories were derived from the categorizations provided by the pharmacy websites themselves. The screenshot of the website of Roadnight Pharmacy from Sidcup in Great Britain in Figure 2 is an example of a pharmacy website with an integrated Web shop. The Web shop offers a range products for sale, including health products such as the wound dressing product at the bottom of the screenshot and nonhealth services such as the hair dye product also at the bottom of the screenshot.

#### Menu and Images

Another part of the coding gauged the combination of clinical and commercial services on (1) the website menu and (2) through website images. The focus thereby was on the homepage as the place where users “land” after opening a pharmacy website.

##### Homepage Menu

The menu on the homepage of a community pharmacy website is the main tool that allows users to navigate to specific services. The menu can list items that connect to clinical services on the website (eg, “prescriptions,” “immunizations”), items that connect to commercial services (“fragrances,” “gifts”), or items that connect to other services (eg, “site map,” “home”). In the screenshot of the website of Medsen Pharmacy from the Netherlands in Figure 3, for instance, the menu at the top of the page has 5 categories. From left to right, these are “About us,” “Health ABC,” “Our services,” “Permission” (to share medical information with other health providers), and finally “Registration.”

To gauge the presence of a combination of clinical and commercial services on the homepage menus, each item on the homepage menu was coded as connecting to a clinical service or a commercial service, using the definitions of clinical and commercial services as given above. A category “other” was used for menu items connecting to other utilities or services such as “contact” or “pharmacy locator.”

##### Homepage Images

Images on community pharmacy homepages are usually either static pictures or sliding pages (“carousel format”). They can serve to highlight clinical pharmacy services, such as a photo of a patient having his blood pressure taken (see example of Hollywood pharmacy in Figure 1). They can also serve to highlight commercial services, such as images of nail polishes (see example of Rexall pharmacy in Figure 4). To gauge the presence of a combination of clinical and commercial services in homepage images, each image on the homepage menu was coded as referring to a clinical service or a commercial service, again using the definitions of clinical and commercial services as given above. A third coding category of “other” was used for images that referred to neither clinical nor commercial services, such as a photo of the pharmacy premises or staff members.

### Data Processing and Analysis

The units of analysis were the community pharmacy websites. Chi-square tests of independence were used to test if the presence of a combination of clinical and commercial services on the community pharmacy websites was contingent on pharmacy types (chain pharmacies and single pharmacies), or country (Australia, Canada, Great Britain, and the Netherlands). Phi coefficients gauged associations between the presence of different categories of services on the websites. Significance levels of *P*<.05 were used for all tests.

## Results

### Clinical and Commercial Services Mentioned on the Websites

#### Clinical Services

An overview of the specific clinical services listed on the community pharmacy websites can be found in Table 2, whereby it should be noted that only clinical services mentioned on more than one website are listed in the table. In line with community pharmacies’ traditional function as a dispensaries of prescription medication, all (N=200) community pharmacy websites made mention of this service. It can also be seen in Table 2 that the most commonly mentioned dispensing service, other than medication dispensary at the pharmacy location, tended to emphasize convenience for patients by presenting options for online ordering of prescription medication refills, multi-dosage packaging, and delivery of prescription medication to patients’ home.

Disease prevention and detection services came as the second most often category of clinical services presented on the community pharmacy websites, with 79.5% (N=159/200) of the websites mentioning specific services of this type. Among these, blood pressure tests, stop smoking counseling, and flu vaccinations were the most frequently mentioned services. Clinical consultation services were presented on less but still a substantial share of 65.0% (N=130/200) community pharmacy websites. The number one specific clinical consultation service thereby was “medication therapy review and management,” which is used here as an umbrella term for a recognized clinical pharmacy service aimed at optimizing therapeutic outcomes through an assessment of a patient’s patterns of medication use, therapeutic responses, and potentially adverse reactions. Depending upon country, the service is known as medication use review, medication therapy management, or home medicines review [56,57]. Consultations for asthma and diabetes patients came second and third, respectively, as the most often mentioned clinical consultation services.

**Table 2:** Presence of clinical services on community pharmacy websites in Australia, Great Britain, Canada, and the Netherlands.

Clinical services^a^			Location of community pharmacies					
			Australia	Great Britain	Canada	Netherlands	All	*P*^b^
**Dispensing services**			50 (100%)	50 (100%)	50 (100%)	50 (100%)	200 (100%)	N/A
	Online request for refill prescription medication^c^		0 (0%)	45 (90%)	30 (60%)	49 (98%)	124 (62.0%)	<.001
	Medication multidosage packaging^d^		21 (42%)	8 (16%)	36 (72%)	26 (52%)	91 (45.5%)	<.001
	Medication home delivery^e^		11 (22%)	14 (28%)	33 (66%)	28 (56%)	86 (43.0%)	<.001
	Medication compounding^f^		10 (20%)	0 (0%)	27 (54%)	4 (8%)	41 (20.5%)	<.001
	Emergency contraception		0 (0%)	27 (54%)	0 (0%)	0 (0%)	27 (13.5%)	<.001
	Online physician for ordering prescription medication^g^		0 (0%)	23 (46%)	0 (0%)	0 (0%)	23 (11.5%)	<.001
	Minor ailment service^h^		1 (2%)	13 (26%)	7 (14%)	0 (0%)	21 (11.0%)	<.001
	Hormone replacement therapy^i^		2 (4%)	0 (0%)	9 (18%)	0 (0%)	11 (5.5%)	<.001
	Opioid substitution^j^		4 (8%)	1 (2%)	2 (4%)	0 (0%)	7 (3.5%)	<.16
**Disease prevention and detection services**			41 (82%)	43 (86%)	37 (74%)	38 (76%)	159 (79.5%)	<.44
	Stop smoking counseling		7 (14%)	30 (60%)	13 (26%)	9 (18%)	59 (29.5%)	<.001
	Travel health advise		4 (8%)	0 (0%)	3 (6%)	20 (40%)	27 (13.5%)	<.001
	Healthy eating and weight advise		10 (20%)	6 (12%)	5 (10%)	2 (4%)	23 (11.5%)	<.09
	Medication use during Ramadan advise		0 (0%)	0 (0%)	0 (0%)	11 (22%)	11 (7.3%)	<.001
	**Diagnostic tests and checks**							
		blood pressure	33	15	14	0	62	
		diabetes/glucose	14	9	5	14	42	
		cholesterol	15	2	2	1	20	
		body mass	12	0	0	0	12	
		bowel cancer	6	2	0	0	8	
		spot check	1	6	0	0	7	
		Chlamydia/gonorrhea	4	1	1	0	6	
		anticoagulation	0	2	0	0	2	
		Allergy	0	2	0	0	2	
	**Vaccinations**							
		flu	15	21	22	0	58	
		travel	0	14	20	0	34	
		shingles	0	0	7	0	7	
**Clinical consultation services**			35 (70%)	36 (72%)	41 (82%)	18 (36%)	130 (65.0%)	<.001
	Medication therapy review and management^k^		20 (40%)	28 (56%)	40 (78%)	2 (4%)	90 (45.0%)	<.001
	**Consultations for patients with specific conditions**		22 (44%)	20 (40%)	20 (40%)	18 (36%)	80 (40.0%)	<.85
		asthma/COPD	11	9	7	14	41	
		diabetes	8	0	13	13	34	
		pain	1	10	5	0	16	
		sleep apnea	11	0	0	0	11	
		cancer	0	4	4	0	8	
		urinary incontinence	1	0	0	7	8	
		post-hospital discharge	3	2	1	1	7	
		hair loss	0	2	1	0	3	
		osteoporosis	0	0	3	0	3	
		coeliac	3	0	0	0	3	
		erectile dysfunction	0	1	1	0	2	
	Absence from work certificates^l^		16 (32%)	0 (0%)	0 (0%)	0 (0%)	16 (8.0%)	<.001

^a^Only clinical services that were mentioned on more than one website are included.

^b^On the basis of the chi-square test of independence *χ*^2^_3_ (N=200): N/A=nonapplicable.

^c^Patients submit an online request for a refill of a medicine that a physician prescribed to them earlier, after which the pharmacy arranges the order for the refill directly with the physician and makes the refill medication ready for the patient.

^d^The pharmacy promises to deliver medication to the patient’s home, or alternatively patients receive a code that will allow them to open a locker where their medication was deposited.

^e^A blister package (“webster pak”) or roll of sachets containing the medications a patient should take on a specific day and time, often used for patients on a multiple medication regime.

^f^The pharmacy prepares a medicine in-house that is tailored to the specific needs of an individual patient.

^g^Patients obtain prescription medication for specific conditions by filing an online questionnaire that is reviewed and then ordered by a physician hired by the pharmacy.

^h^Pharmacist advises and prescribes medicine for common, non–life threatening conditions such as eczema, headaches, or coughs.

^i^A therapy for menopause-related symptoms involving medications to artificially boost hormone levels.

^j^Substitution medication for patients with an opioid dependence, including the dispensing of methadone.

^k^Consultation to review a patient’s medication use to check for possible overlaps or interactions, identify and diminish side effects, and improve a patient’s understanding and medication adherence.

^l^Pharmacist issues a certificate as proof of legitimate absence from work, part of the 2009 Australian Fair Work Act.

**Table 3:** Presence of commercial services on community pharmacy websites in Australia, Great Britain, Canada, and the Netherlands.

Commercial services		Location of community pharmacies					*P*^a^
		Australia	Great Britain	Canada	Netherlands	All	
**Health-related services**		25 (50%)	31 (62%)	44 (88%)	25 (48%)	119 (59.5%)	<.001
	Over-the-counter medicine^b^	30 (60%)	31 (62%)	29 (58%)	25 (50%)	115 (57.5%)	ns
	Complementary and alternative medicine	24 (48%)	30 (60%)	29 (58%)	24 (48%)	107 (53.5%)	ns
	Home medical aids^c^	33 (66%)	19 (38%)	24 (48%)	22 (44%)	98 (49.0%)	.03
	Diet products^d^	28 (56%)	27 (54%)	17 (34%)	22 (44%)	94 (47.0%)	ns
**Nonhealth-related services**		32 (76%)	30 (60%)	32 (88%)	32 (64%)	126 (63.0%)	ns
	Cosmetic products	32 (64%)	30 (60%)	32 (64%)	32 (64%)	126 (63.0%)	ns
	Foods and beverages	0 (0%)	5 (10%)	13 (26%)	6 (12%)	24 (16.0%)	<.001
E-commerce		20 (40%)	30 (60%)	9 (18%)	32 (64%)	91 (45.5%)	<.001

^a^On the basis of the chi-square test of independence *χ*^2^_3_ (N=200): ns=nonsignificant.

^b^Products dispensing of some of the specific classes of over-the-counter drugs can be restricted to pharmacists or drug store owners only. This concerns only a very small portion of the wide range of over-the-counter medicines available.

^c^Tools to mitigate medical treatment or impairment, such as mattress covers, crutches, or shower seats. Usually sold, but sometimes also for hire, through the pharmacy.

^d^Products that replace or complement conventional foods, taken for health or cosmetic reasons, such as slimming shakes, vitamins, and gluten-free foods.

#### Commercial Services

The overview of the commercial services presented on the community pharmacy websites in Table 3 shows that nearly 60% of the community pharmacy websites (N=119/200, 59.5%) presented the sale of health products on their websites. Particularly the sale of over-the-counter medicine such as cold and cough medicine, as well as complementary and alternative medicine such as homeopathic products, were popular items on the community pharmacy websites. The sale of nonhealth services was even more common on the websites (N=126/200, 63.0%). Commonly mentioned in this category of services was the sale of cosmetic products such as shampoos or hand creams; however, the offer of nonhealth-related services on the pharmacy websites could also extend into a very wide range of goods or services, including public transport and lottery tickets, walking sticks, computers, or an organic juice bar inside the pharmacy. Finally, nearly half (N=91/200, 45.5%) of the pharmacy websites included e-commerce options (ie, a Web shop) for the online sale of commercial products.

#### Cross-National Differences

Although cross-national comparisons were not this study’s primary aim, the last columns in Tables 2 and 3 show the extent to which the mention of specific clinical and commercial services on the community pharmacy websites was contingent upon the country where the pharmacy is located, based on chi-square tests of independence. A notable finding here was that there were few differences across countries in the categories of clinical services mentioned on the community pharmacies websites. Both dispensing services and disease detection and prevention services were mentioned equally often on the community pharmacy websites across countries. On the other hand, a rather large share of the specific clinical services within these categories proved country-specific. Thus, many specific clinical services, be it medication compounding, consultations for diabetes patients, or stop smoking counseling services, were mentioned on the pharmacy websites in one country significantly more often than in other countries. Furthermore, a notable cross-national difference in the mention of commercial services on websites was the relatively low share of Canadian pharmacies that integrated e-commerce in their websites compared with the three other countries.

**Table 4:** Presence of services on chain pharmacy websites and single pharmacy websites.

Presence of services		Pharmacy business type		
		Chain pharmacy (N=147)	Single pharmacy (N=53)	*P*^a^
**Clinical services**				
	Dispensing services	147 (100%)	53 (100%)	N/A
	Clinical consultation services	101 (68.7%)	30 (57%)	ns
	Disease prevention and detection services	120 (81.6%)	31 (59%)	<.001
**Commercial services**				
	Health-related commercial services	104 (70.7%)	22 (42%)	<.001
	Nonhealth-related commercial services	114 (77.6%)	13 (25%)	<.001
	E-commerce	85 (57.8%)	7 (13%)	<.001

^a^On the basis of the chi-square test of independence *χ*^2^_1_ (N=200): N/A=nonapplicable, ns=nonsignificant.

#### Differences Between Single and Chain Pharmacies

Table 4 shows that chain pharmacy websites presented disease prevention and detection services as well as all categories of commercial services significantly more often than single pharmacy websites. Note that this does not mean that single pharmacy websites did not mention these services, as all categories of clinical services were mentioned on more than half of the single pharmacy websites. Additionally, health-related commercial services such as self-care products were present on 42% (22/53) of the single pharmacy websites in the sample compared with 70.7% (104/147) of the chain pharmacy websites.

### Combinations of Clinical and Commercial Services

The analyses focusing on a combination of clinical and commercial services on community pharmacy websites showed that nearly three quarters (N=145/200, 72.5%) of the community pharmacy websites were a mix of clinical and commercial services. The remaining pharmacy websites presented clinical services only (N=55/200, 27.5%). This confirms that having a combination of clinical and commercial services on the website of licensed community pharmacies is widespread. Although the share of pharmacy websites containing a combination of clinical and commercial services was at least 62% (31/50) in each country, a chi-square test of independence further showed that combinations were not equally common across the 4 countries, *χ*^2^_3_ (N=200)=10.91, *P*=.01. They were most common on the Canadian community pharmacy websites (N=44/50, 88%), followed by the Australian (N=38/50, 76%), British (N=32/50, 64%), and finally the Dutch (N=31/50, 62%) community pharmacy websites. Moreover, whereas more than 4 in every 5 websites from chain pharmacies contained a combination of clinical and commercial services (N=120/147, 82.8%), slightly less than half of the single pharmacy websites did (N=25/53, 47%), resulting in a significant chi-square relationship (*χ*^2^_1_ (N=200)=24.12, *P*<.001). A combination of clinical and commercial services on community pharmacy websites was thus more commonly seen on the websites of pharmacies operating under a chain brand than single pharmacies.

Table 5 renders phi coefficients for the degree of association between the presence of specific categories of clinical services and specific categories of commercial services on the community pharmacy websites. It can be seen that specifically the mention of disease prevention and detection services (eg, stop smoking counseling, flu vaccinations, diabetes tests) was significantly associated with the mention of commercial services on the websites. The mention of dispensing services, on the other hand, was unrelated to the mention of commercial services on the websites, whereas there was a significant negative association with the presence of e-commerce services.

**Table 5:** Phi coefficients for the presence of categories of clinical and categories of commercial services on the community pharmacy websites (ϕ, 200).

Clinical services	Commercial services			
	Health-related	Nonhealth-related	E-commerce	All commercial services
Dispensing services	.09	.01	−.17^a^	.00
Consultation services	.19^b^	.00	−.15^a^	.09
Disease prevention and detection services	.14^a^	.26^b^	.11	.26^b^

^a^*P*<.05.

^b^*P*<.01.

### Menu and Images

#### Homepage Menu

One of the main elements of community pharmacy websites is the homepage menu that allows users to navigate to specific services on the website. On nearly half of the pharmacy websites’ homepages, namely 99/200 homepages (49.5%), the homepage menu was a combination of clinical and commercial service items. The occurrence of this combination of clinical and commercial services on the homepage menus was further contingent on country and occurred most often on the Canadian websites (37/50, 74%), followed by the British (N=30/50, 60%), Australian (N=24/50, 48%) and finally the Dutch (N=6/50, 12%) websites, *χ*^2^_3_ (N=200)=42.71, *P*<.001. Also, a combination of clinical and commercial homepage menu items were significantly more common on chain pharmacy websites (N=82/147, 55.8%) than single pharmacy websites (N=15/53, 28%), *χ*^2^_1_ (N=200)=11.75, *P*<.001.

#### Homepage Images

Another way of combining clinical and commercial content on the homepages of the community pharmacies’ websites is through the inclusion of clinical and commercial images on the homepage. More than half of the homepages of community pharmacy websites contained a combination of clinical and commercial images (N=107/200, 53.5%). A combination of clinical and commercial images occurred most often on the homepages of the British pharmacy websites (N=40/50, 80%), followed by the Australian (N=25/50, 50%), Canadian (N=24/50, 48%), and finally the Dutch (N=24/50, 48%) homepages, *χ*^2^_3_ (N=200)=21.12, *P*<.001). Again, it was significantly more common on the homepages of chain pharmacy websites (N=97/147, 66.0%) than on the homepages of single pharmacy websites (N=10/53, 19%), χ^2^_1_ (N=200)=35.40, *P*<.001).

## Discussion

### Principal Findings

Community pharmacy websites are playing an increasingly important role in clinical health care nowadays as they have moved from a static reflection of local premises for dispensing medication to an online “health care hub.” Here, patient consumers can submit electronic prescriptions, order prescription and over-the-counter medication, consult pharmacists on the management of a disease, download software to monitor one’s health, and order vaccinations, personal care, and household products, among other things. Pharmacy licensing however lags behind these developments and often does not include specific regulations about a combination of clinical and commercial services on pharmacy websites. The present study is situated amidst these developments, which may be a significant “social force” as online retail continues to grow but has not received much attention in the literature so far in the context of pharmacy websites [47-50]. This study addressed the pertinent question as to what types of clinical and commercial services are mentioned on present-day licensed community pharmacy websites and to what extent the websites currently present a combination of clinical and commercial services. Such a combination is regarded as highly controversial in the clinical professions and tends to frequently raise calls for codes of conduct, regulatory action, or straightforward bans to protect vulnerable patient groups from the undue influence on their preferences for treatment [1-3].

Through a content analysis of representative samples of licensed community pharmacy websites in Great Britain, the Netherlands, Australia, and Canada, it was found that around three quarters of the websites of licensed community pharmacies currently present a combination of clinical and commercial services. Particularly disease prevention and detection services such as stop smoking counseling and travel health advice were often combined with the sale of commercial services on the websites. This shows, first, that a controversial combination of clinical and commercial services is a common feature of licensed community pharmacy websites nowadays. Furthermore, particularly the inclusion of disease prevention and detection services on the pharmacy websites signals an appeal to a broad group of customers to maintain their health through a combination of timely disease detection and prevention and purchasing health and wellness products such as over-the-counter medicine, diet shakes, and aromatherapy oils.

Up to three quarters of community pharmacies in our sample operate under a chain pharmacy’s retail brand name, and the findings showed that a combination of clinical and commercial services is particularly common on chain pharmacies’ websites, although also nearly half of the single pharmacy websites included a combination of clinical and commercial services. One explanation is that commercial content for chain pharmacy websites is created in the head offices, whereas single pharmacy owners may not have similar resources. Another explanation may be that single pharmacy owners attempt to avoid the controversial combination of clinical and commercial contents on their websites more than chains in an attempt to emphasize adherence to the trade’s traditional values and present as different from the chain pharmacy competition in this respect. Single community pharmacies have been found to prioritize the profession’s fiduciary role more than chain pharmacies [16,58].

A final notable finding from this study is that there were few differences across countries in the categories of clinical services mentioned on the community pharmacies websites, and both dispensing services and disease detection and prevention services were mentioned equally often on the community pharmacy websites across countries. This suggests that community pharmacy websites generally include the same scope of services across the four high-income countries. However, there was substantial variation within, as well as across, countries in regard to specific clinical services mentioned on the community pharmacy websites. This can in part be explained by the specific national context. For instance, online pharmacies that are (actually or supposedly) “Canadian” obtained a negative reputation in recent years for allegations of illegitimate e-commerce practices [59]. It appears that licensed community pharmacies from Canada therefore more often did not include e-commerce options than other countries. Other cross-national differences can be explained by national pharmacy legislation. For instance, receiving a prescription from an online physician through a community pharmacy website occurred only on British pharmacy websites because it is prohibited in the three other countries in our sample [60], while vaccinations through pharmacies, for instance, are not allowed in the Netherlands [61]. The larger share of the cross-national variation however cannot be explained by nation-specific context or legislation, suggesting that the community pharmacies’ offerings are not only shaped by clinical considerations, but also by other factors. Possible factors include insurer-based remuneration programs [62], as well as government- and industry-sponsored health and disease campaigns [1,63].

### Limitations

Regarding the generalizability of the findings, it is important to note that the present study endeavored to gain insight into the combination of clinical and commercial communication on present-day community pharmacy websites. No claim regarding a one-to-one relationship with the actual services offered at a pharmacy’s premises is made, as this falls outside this study’s aims. It is of further relevance to note that the community pharmacy websites in our sample likely render a somewhat conservative picture of the combination of clinical and commercial content on community pharmacy websites worldwide. First, because the focus of this study was on websites of licensed community pharmacies, rather than the more heavily studied phenomenon of online pharmacies involved in illicit practices [10-12]. Second, regulations surrounding commercial communication in health care are typically enforced more heavily in the countries in our sample than in middle- or low-income countries [53]. Third and lastly, mass merchants and supermarkets with pharmacy departments were excluded from the present sample since these websites would not allow a clear separation between services mentioned as part of pharmacy departments, versus other store departments. The websites of some of the largest mass merchants in the world such as Wal-Mart, Tesco, and Costco frequently have pharmacy sections as well, which leads to the combination of clinical services with a vast range of commercial services. Although caution is warranted due to a lack of empirical data, the combination of clinical and commercial communication found in this study likely forms a lower bound, rather than an upper bound, of those found on community pharmacy websites across the board.

### Implications

This paper has drawn attention to the websites of licensed community pharmacies as one of the major online venues in health care where a combination of clinical and commercial services is common practice. One implication is a further recognition in the literature on online DTCA and medical marketing that this is an important area of health care communication on the Internet nowadays. Effects on online patients’ reactions have yet to be studied but, as mentioned in the introductory section, patients often see online health information as more credible and persuasive when it comes from a source that is perceived as having relevant expertise [30-33]. With the increasingly expanding role of community pharmacies in clinical health care provision, this suggests for policy makers and regulatory agencies that the combination of clinical and commercial services on community pharmacy websites is able to impact patients’ beliefs and preferences. It should thereby be noted that, likening to contemporary advertising formats such as sponsored content and native advertising [64,65], the distinction between services restricted to licensed pharmacists versus commercial services is nearly never clearly marked on community pharmacy websites. Due to this, patients cannot immediately tell if a service or product, such as a magnetic therapy service or insect repellent, featured on a community pharmacy website is clinical or commercial. The extant research shows that people are rarely motivated, or able, to distinguish commercial content from editorial content in the absence of clear disclosures [65,66]. All in all, community pharmacy websites clearly are no longer just an online version of a medication dispensing facility alone and should be regarded as a significant party in the controversy over combinations of clinical and commercial communication on the Internet nowadays.

## Abbreviations

COPD: chronic obstructive pulmonary disease
DTCA: direct-to-consumer advertising
FTC: American Federal Trade Commission
